# Research on the measurement of intracranial hemorrhage in rabbits by a parallel-plate capacitor

**DOI:** 10.7717/peerj.10583

**Published:** 2021-01-05

**Authors:** Zelin Bai, Haocheng Li, Jingbo Chen, Wei Zhuang, Gen Li, Mingsheng Chen, Jia Xu, Shuanglin Zhao, Yuening Liu, Jian Sun, Feng Wang, Lin Xu, Mingxin Qin, Gui Jin

**Affiliations:** 1 College of Biomedical Engineering, Third Military Medical University (Army Medical University), Chongqing, China; 2 School of Pharmacy and Bioengineering, Chongqing University of Technology, Chongqing, China

**Keywords:** Parallel-plate capacitor, Permittivity, Intracranial hemorrhage, Capacitance

## Abstract

Intracranial hemorrhage (ICH) carrying extremely high morbidity and mortality can only be detected by CT, MRI and other large equipment, which do not meet the requirements for bedside continuous monitoring and pre-hospital first aid. Since the biological tissues have different dielectric properties except the pure resistances, and the permittivity of blood is far larger than that of other brain tissues, here a new method was used to detect events of change at the blood/tissue volume ratio by measuring of the head permittivity. In this paper, we use a self-made parallel plate capacitor to detect the intracranial hemorrhage in rabbits by contactless capacitance measurement. The sensitivity of the parallel-plate capacitor was also evaluated by the physical solution measurement. The results of physical experiments show that the capacitor can distinguish between three solutions with different permittivity, and the capacitance increased with the increase of one solution between two plates. At the next step in the animal experiment, the capacitance changes caused by 2 ml blood injection into the rabbit brain were measured. The results of animal experiments show that the capacitance was almost unchanged before and after the blood injection, but increased with the increase of the blood injection volume. The increase of capacitance caused by blood injection was much larger than that before and after blood injection (*P* < 0.01). The experiments show that this method is feasible for the detection of intracranial hemorrhage in a non-invasive and contactless manner.

## Introduction

Spontaneous intracranial hemorrhage refers to the hemorrhage caused by the rupture of blood vessels in the brain parenchyma. It is the most serious type of acute stroke because of its emergency, dangerous condition, high morbidity and mortality. In Europe and the United States and other Western developed countries, spontaneous intracranial hemorrhage accounted for about 10% to 20% of all stroke patients. Taking the United States as an example, it is reported that the mortality rate of cerebral hemorrhage is 23% ∼52% (Shehadah, Franklin & Benson, 2016). According to statistics of the China Stroke Prevention and Treatment Report 2018, the incidence rate of hemorrhagic stroke was 126.34 per 100,000 person-years (Wang et al., 2019). In the clinic, we can only rely on imaging instruments like CT and MRI to detect intracranial hemorrhage. These equipment are bulky, expensive, demanding, unable to achieve bedside monitoring, unable to apply to acute hemorrhage and critical patients, and unable to play a role in pre-hospital emergency. Therefore, a portable, low-cost and fast detection technology of intracranial hemorrhage is urgently needed.

Because different biological tissues have different impedance characteristics, the same biological tissue also has different impedance characteristics under different pathophysiological conditions, it is feasible to detect intracranial hemorrhage by measuring the impedance changes (Mcadams & Jossinet, 1995). Electrical impedance tomography (EIT) and magnetic induction tomography (MIT) are two of the most typical electrical impedance imaging technologies, which have the advantages of non-destructive, harmless, fast response, low cost and functional imaging (Bayford, 2006). They are the most likely new technologies to meet the above requirements. EIT injects a weak current into the electrode on the human body surface, and measures the voltage value on other electrodes. According to the relationship between voltage and current, it reconstructs the absolute value or variation of the internal impedance of the human body. EIT has been used in lung function imaging, breast cancer imaging, gastric emptying, epilepsy and brain nerve activity detection (Adler & Boyle, 2017). The research group of Holder professor at University College London (UCL) has been committed to the detection of EIT in stroke for a long time, and has achieved rich research results (Yerworth et al., 2002). Dowrick, Blochet & Holder (2015) found that at low frequency, the conductivity of ischemic tissue was 10–20% lower than that of normal brain tissue, and the conductivity of blood was higher than that of normal brain tissue. According to the principle of electromagnetic induction, MIT uses the coil to generate AC magnetic field through the measured head, measures the disturbance of the induced field to the excitation field, extracts the conductivity information of the measured brain tissue, and reconstructs the conductivity distribution (Griffiths, 2004). Because the AC magnetic field can easily pass through the skull and its attenuation is very little in the brain tissue, MIT is a real non-contact and non-invasive measurement. MIT and EIT are both used to measure the electrical impedance of the measured object, the only difference is the measurement medium, the former uses AC magnetic field and the latter uses AC current. In 2013, based on the method of magnetic induction, Gonzalez et al. (2013) measured the changes of magnetic induction phase shift of 46 healthy volunteers and eight patients with CT-confirmed brain edema and brain hematoma within 1–200 MHz and found that the measurement results of different frequency intervals can be used to distinguish the healthy volunteers, intracranial hemorrhage (ICH) patients and brain edema patients. Oziel et al. (2016) used the same method as Gonzalez et al. to detect the cerebrovascular autoregulation. Kellner et al. (2018) designed a symmetrical volumetric impedance phase shift spectroscopy (VIPS) device based on the symmetrical characteristics of the left and right hemispheres of the brain. The phase shift of magnetic induction disturbance was measured in different frequency bands to detect and classify stroke. And good results were obtained in clinical experiments (Kellner et al., 2018). In 2018 and 2019, Oziel and colleagues designed a single coil structure, using broadband measurement data to detect stroke, and achieved good results (Oziel et al., 2020; Oziel, Korenstein & Rubinsky, 2019; Oziel, Korenstein & Rubinsky, 2020). In addition to EIT and MIT, microwave imaging has been used to detect stroke in recent years. The microwave technique depends on the existence of a significant dielectric contrast between blood and other tissues. Mobashsher & Abbosh (2016) designed a portable noninvasive multi slice wideband head imaging system. The hematoma model with the size of 2*2*0.5 cm^3^ in realistic head phantoms can be found in physical imaging experiments. In addition, the electromagnetic coupling method can also be used for monitoring of cardiovascular activity (Teichmann et al., 2013).

At present, EIT only measures the real part information of impedance (pure resistance), only focuses on the conduction of current in extracellular fluid, so the frequency of excitation current used is relatively low (<100 kHz). Low frequency current reduces the difficulty of impedance measurement, but high impedance skull greatly attenuates the voltage change caused by the change of intracranial impedance and reduces the sensitivity of brain tissue measurement. Studies have shown that when the impedance of simulated stroke tissue changes 100–200%, the corresponding boundary voltage changes only 1–2% (Horesh, 2006). Due to the limitation of magnetic induction measurement theory, MIT only imaged the imaginary part (including conductivity parameter) of the induced field relative to the excitation field. Although the real part of the induced field relative to the excitation field contains the information of the permittivity, it is very difficult to detect because the displacement current generated by the electric field is too small, and the conductivity of the measured object must meet certain conditions, so few people pay attention to the real part. The conductivity of biological tissue is very low (0.1 s/m ∼2 s/m), and the induced magnetic field is very weak. Some studies have shown that when the working frequency is 10 MHz, it needs a phase measurement accuracy of 0.01° to detect the phase shift of the induced field relative to the excitation field in biological tissues, which makes it very difficult for MIT brain imaging (Watson et al., 2001).

The biological tissue cell membrane is similar to the incomplete small capacitor, which is a kind of selective through membrane. It is considered as a dielectric when DC or low-frequency driving current is applied, and has significant dielectric properties. Therefore, the low-frequency current cannot pass through the cell membrane, only through the extracellular fluid. At a high frequency, the distributed capacitance between cell membranes can conduct current, which is capacitive reactance, and the high frequency current can pass through the intracellular fluid (Mcadams & Jossinet, 1995). Therefore, in addition to the different resistance characteristics, the biological tissues also show different capacitive reactance characteristics, which are different permittivity. The permittivity of blood is much higher than that of other brain tissues. At 1 MHz, the permittivity of blood, gray matter and cerebrospinal fluid are 3,000, 990,108, respectively (Gabriel, Lau & Gabriel, 1996). Therefore, when intracranial hemorrhage occurs, the permittivity of the whole head will increase significantly. It is theoretically feasible to detect intracranial hemorrhage by measuring the permittivity of the head. Griffiths et al. (2007) measured the real part of the induced field relative to the excitation field induced by normal saline, and deduced that the permittivity of normal saline was 87 ± 6, which is very close to that of the known value of water.

Our research group has been studying the magnetic induction detection technology of brain for a long time. We have used the method of magnetic induction coupling to measure the intracranial hemorrhage in rabbits (Jin et al., 2014; Yan et al., 2016) based on brain conductivity measurements. However, it is better to measure the permittivity, since at the early stage of ICH, the CSF starts a compensatory mechanism. With increase of hematoma volume, CSF is slowly discharged, and since the conductivity of CSF is twice that of blood the overall head conductivity slowly drops. After the CSF is depleted, the overall head conductivity slowly rises with the increase of hematoma. Thus, with the rising amount of bleeding, the overall head conductivity neither linearly increases nor is directly decided by the amount of bleeding (Yan et al., 2016). This situation is unfavorable for accurately measuring the amount of bleeding. Moreover, since the permittivity of blood is far larger than those of CSF and other brain tissues, the overall head permittivity always gradually increases with the rising amount of bleeding. At the compensatory stage, when an equal volume of blood discharges an equal volume of CSF, the overall head permittivity is still on the rise. Therefore, it is more appropriate to detect the intracerebral hemorrhage by measuring the permittivity of the head. For the detection of magnetic induction coupling, the permittivity parameter is included in the real part of the induced field relative to the excitation field. In the last article (Bai et al., 2019), we used the same magnetic induction coupling method to measure the real part changes of the induced field relative to the excitation field caused by intracranial hemorrhage in rabbit head. The experimental results show that the real part changes increased with the increase of blood injection (Bai et al., 2019). It is proved that it is feasible to detect intracranial hemorrhage by measuring the change of permittivity. As a result of the real part of the magnetic induction disturbance is very small, it is very difficult to measure, and it is limited by many conditions. This paper attempts to use a parallel-plate capacitor to directly measure the capacitance of the head with intracranial hemorrhage, and uses the measured changes of capacitance to reflect the changes of hemorrhage volume. In recent years, capacitive sensors have been used to measure the nerve action potential of brain, including detection of somatosensory evoked potentials, auditory evoked potential and so on (Darmawan et al., 2017; Taruno et al., 2014). There are also studies on the use of capacitive sensors to detect intracranial tumors and other foreign bodies. To the best knowledge of the authors, all of these studies have not progressed beyond the phase of examining the physical model (Taruno et al., 2013; Ibrahim et al., 2017). This paper attempts to use parallel plate capacitor to detect intracerebral hemorrhage, which is also the first attempt of animal experiment. The article is made up of two parts: in the first part (the physical model), we present a capacitance measurement system capable of distinguishing between three different permittivity solutions; in the second part, we tested the system in an animal experiment, and it seems that the capacitance measured with the system increases approximate linearly with the increase of blood volume.

## Materials & Methods

### Head capacitance measurement theory

**Figure 1:**
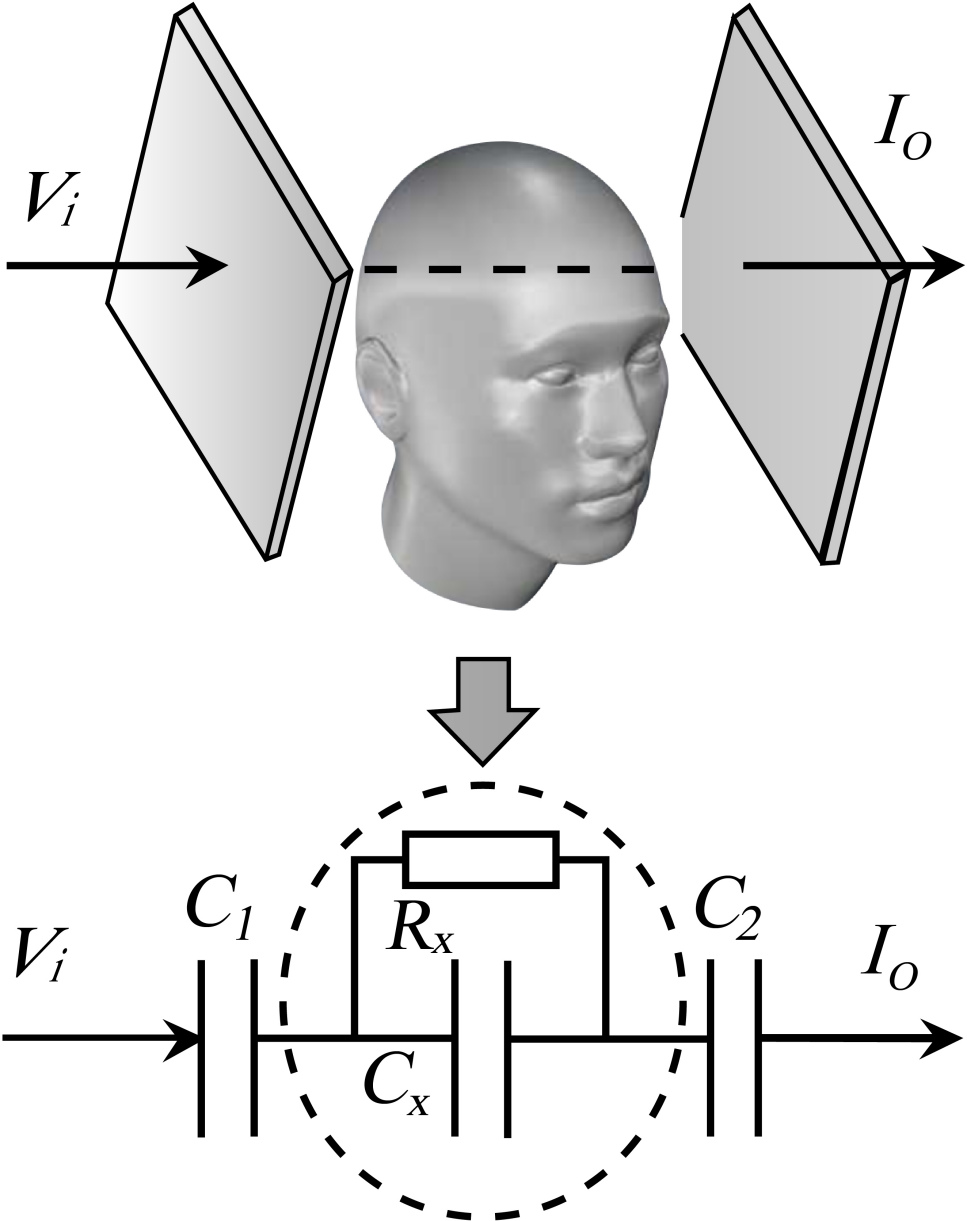
The equivalent measurement model of head with a parallel-plate capacitor.

According to the Cole-Cole model of biological tissue and the principle of high frequency capacitance coupling, a head capacitance measurement model as shown in Fig. 1 is proposed. *C*_1_ and *C*_2_ are the equivalent capacitance between the capacitor plate and the skull, *C*_*x*_ is the equivalent capacitance of brain tissue, and *R*_*x*_ is the equivalent resistance of brain tissue. The total capacitance *C*_*total*_ between the two plates satisfies Eq. (1), where ε_*r*_ is the equivalent permittivity of the head between the two plates, ε_0_ is the vacuum permittivity, *S* and *d* are the surface area of the plate and the distance between the two plates respectively. According to Fig. 1, when the measuring position remains unchanged, *C*_1_ and *C*_2_ remain unchanged, the total capacitance *C*_*total*_ between the two plates is only related to *C*_*x*_, that is, only to the equivalent permittivity of the measured head. When intracranial hemorrhage occurs, the head equivalent permittivity increases, *C*_*x*_ increases.

(1) \begin{eqnarray*} \frac{1}{{C}_{total}} \approx \frac{1}{{C}_{1}} + \frac{1}{{C}_{2}} + \frac{1}{{C}_{x}} \rightarrow \Delta C\propto {C}_{x}= \frac{{\varepsilon }_{r}\cdot {\varepsilon }_{0}\cdot S}{d} \end{eqnarray*}

### Parallel-plate capacitance measurement system

**Figure 2:**
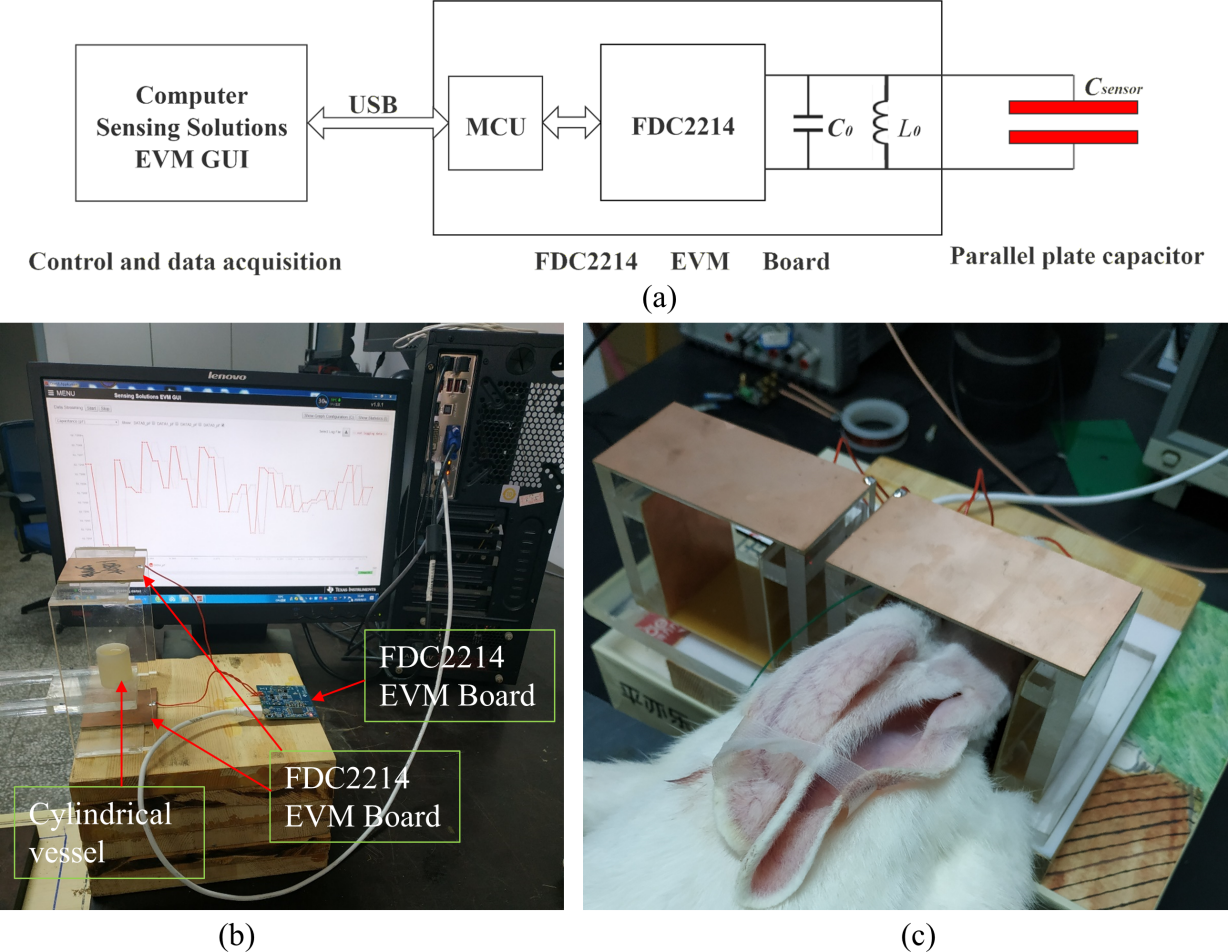
(A) Principle block diagram of measurement system. (B) Physical experiment system. (C) Animal experiment system.

The principle of the parallel plate capacitance measurement system is shown in Fig. 2A, and the photo of the system is shown in Fig. 2B. The parallel plate capacitor consists of two identical parallel copper clad laminates. Electrode plate is cut with epoxy glass fiber copper clad panel, and the thickness of the base plate is two mm. During measurement, the measured object is placed in parallel between two plates. This paper uses FDC2214 capacitance measuring chip (Ti) to measure the capacitance of the parallel-plate capacitor (Texas Instruments, 2015). The chip measures the total capacitance in parallel to the two ports of one channel and directly converts the measured capacitance into digital signal output. The specific measurement tool is the FDC2214 EVM development board of Texas Instruments (Texas Instruments, 2016). This development board mainly integrates a FDC2214 chip and a MSP430 MCU. MSP430 communicates with the computer through a USB cable. On the one hand, it receives the control command from the computer, sets the parameters of FDC2214 chip and controls its work; on the other hand, it uploads the capacitance data measured by FDC2214 to the computer. A FDC2214 data acquisition software (sensing solutions EVM GUI, Ti) is used to collect and display the measured data of FDC2214. FDC2214 EVM board has a channel port for connecting external capacitor, which is easy to use. Therefore, we directly welded the parallel plate capacitor to the measurement port of FDC2214 EVM board through wires. FDC2214 EVM board and data acquisition software are shown in Fig. 2B.

FDC2214 needs to connect a reference inductance and capacitance between two pins of the channel to form a parallel LC circuit, as the *L*_0_ and *C*_0_ shown in Fig. 2A. The measured external parallel plate capacitor *C*_*sensor*_ is also connected to the channel pins. So *L*_0_, *C*_0_, *C*_*sensor*_ constitute a LC parallel circuit. FDC2214 calculates the *C*_*sensor*_ by measuring the resonant frequency f of the LC parallel circuit. Assuming that the resonant frequency is *f* and the total capacitance of the parallel circuit is *C*, then *C* satisfies Eq. (2). Since *L*_0_ and *C*_0_ are known, the capacitance *C*_*sensor*_ of the external parallel plate capacitor is shown in Eq. (3).

(2) \begin{eqnarray*}C& = \frac{1}{{L}_{0}\cdot (2\pi \cdot f)^{2}} \end{eqnarray*}

(3) \begin{eqnarray*}{C}_{sensor}& =C-{C}_{0}\end{eqnarray*}

When the EVM board leaves the factory, the values of welded *L*_0_ and *C*_0_ are 18 µH and 33pF, respectively. The frequency is about 6.5 MHz when there is no load (the parallel-plate capacitor is not connected). The resonant frequency range supported by FDC2214 is 10 kHz–10 MHz. The capacitance measurement accuracy of FDC2214 is about 10 fFfor the resonance frequency. The measurement speed of FDC2214 is related to the internal sampling time setting, which is about 15 times per second in this paper. The measurement system of physical experiment and animal experiment is shown in Figs. 2B and 2C. The size of the parallel plate used in physical experiment is 60 mm * 60 mm, and the distance between the two plates is 120 mm. The size of the parallel plate for animal experiment is 100 mm * 50 mm, and the distance between the two plates is 70 mm. The area and spacing of parallel plate capacitors have great influence on the measurement sensitivity. The larger the area of the parallel plate capacitor, the higher the sensitivity, and the smaller the spacing, the higher the sensitivity. In the physical experiment, because the sensitivity of different positions between the two plates is to be measured, and the height of the measured object is 30 mm, the distance between the two plates needs to be larger, and the final setting is 120 mm. According to the values of *L*_0_ and *C*_0_, plus the parallel plate capacitance and stray capacitance caused by wire, the measured resonance frequency of physical experiment system is about 5.2 MHz. In animal experiments, in order to improve the measurement sensitivity, the distance between the two plates should be as small as possible. In this paper, experimental animal is the rabbit. Considering that the height of rabbit’s head is close to 60 mm, the distance between the two plates is set as 70 mm. The area of the plate is just the area covering the rabbit’s head. In addition, the higher the resonant frequency, the higher the sensitivity. Therefore, in order to improve the sensitivity of intracerebral hemorrhage measurement, the original capacitance *C*_0_ of 33pF on the EVM board was replaced by a capacitance of 10pF, which can improve the resonance frequency. According to the values of *L*_0_ and *C*_0_, plus the parallel plate capacitance and stray capacitance caused by wire, the measured resonance frequency of the animal experiment system is about 9.2 MHz. The capacitance *C*_0_ for physical experiment is still 33pF.

### Physical experiment

**Figure 3:**
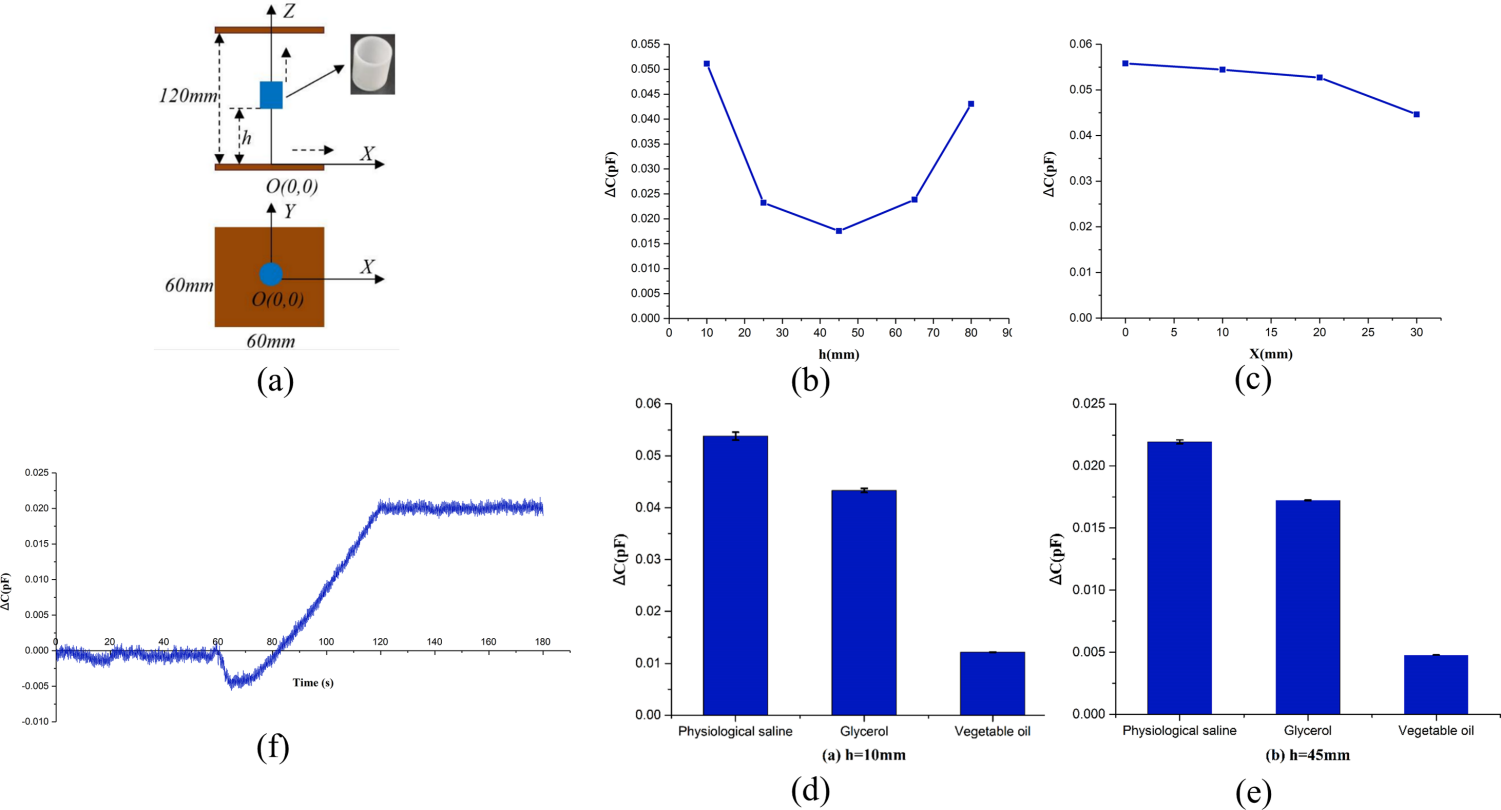
Schematic diagram of physical experiment and the result of physical experiment. (A) Schematic diagram of physical experiment. (B) Capacitance difference of physiological saline and vegetable oil at different heights on *Z*-axis. (C) Capacitance difference of physiological saline and vegetable oil at different positions along *X*-axis. (D) Capacitance change caused by the same volume of three different solutions at Position 1 (*h* = 10 mm). (E) Capacitance change caused by the same volume of three different solutions at Position 2 (*h* = 45 mm). (F) Relationship between capacitance change and injection volume of saline.

The physical experiment is divided into the following three parts: 1. Sensitivity distribution of different positions in horizontal and vertical directions in two plates. 2. Measurement of different permittivity solutions. 3. Continuous measurement during volume increase of physiological saline. The schematic diagram of physical measurement is shown in Fig. 3A. The center of the plate is the coordinate origin *O* (0,0,0), the *XOY* plane is parallel to the plate, and the *XOZ* plane is perpendicular to the plate. All the test solutions were put into 3D printing cylindrical vessels separately. The Cylindrical vessel is shown in Fig. 2B, and is shown in blue in the schematic diagram. The height of the vessel is 30 mm, the inner diameter is 22 mm, and the material is resin. The distance between the bottom of the vessel and the lower plate is *h*.

Experiment 1: 9ml of physiological saline (0.9%) and vegetable oil solution were put into two identical cylindrical vessels described above, separately. One by one, put two vessels containing the tested solution into the same position between two plates to measure the capacitance, and calculated the difference of capacitances of the two solutions at that position. For each position, the measurement was repeated four times and the data was averaged. First, measured the capacitance difference of two solutions at five positions (*h* = 10 mm, 25 mm, 45 mm, 65 mm, 80 mm) along the *Z*-axis. When measuring, the vessel was coaxial to the *Z* axis. Secondly, measured the capacitance difference of two solutions at four positions (*x* = 0 mm, 10 mm, 20 mm, 30 mm; *Z* = 10mm) along the *X*-axis. Experiment 2: 9 ml of physiological saline, glycerin and vegetable oil were respectively put into three identical cylindrical vessels. Another same vessel was used for empty measurement. The four vessels are identical in size and material. The three vessels with solution and another empty vessel were measured at the same position. The measured capacitance value of the vessel with each solution was subtracted by the empty vessel value to obtain the capacitance change caused by each solution. Two positions (*h* = 10 mm (near the plate) and *h* = 45 mm (in the middle between two plates)) on the *Z*-axis were measured. The measurement was repeated four times for each position and the average capacitance change for each solution was calculated. Experiment 3: put an empty vessel coaxially to the *Z*-axis and placed it in the middle position between the two plates (*h* = 45 mm). Injected 9ml of physiological saline into the empty vessel at a constant speed, and measured the capacitance continuously. The injection time was 1 min. Before injection, the empty vessel was measured at rest for 1 min. After injection, the vessel was measured at rest for another 1 min. The total measurement time was 3 min together. The relationship between the measured capacitance and the volume of the solution injected was obtained. The permittivity of three solutions of physiological saline (0.9%), glycerin and vegetable oil are 80, 37, 2–4 respectively, according to relevant literatures.

### Animal experiments

Fifteen New Zealand white rabbits (2.0–2.5 kg in weight) were purchased from the animal center of the Third Military Medical University. Rabbits were arrived one day before experiments and housed under ambient conditions (22 °C, 50% relative humidity, and a 12-h light/dark cycle), with free access to water and chow. These experiments were conducted under the guidance of the Administration of Animal Experiments for Medical Research Purpose issued by the Ministry of Health of China. The protocol was reviewed and approved by the Animal Experiments and Ethical Committee of Army Medical University (AMU, Chongqing, China). All procedures were conducted while minimizing the suffering of rabbits. A model of intracerebral hemorrhage was established by injecting autologous blood into the rabbit brain. The capacitance of the rabbit’s head was measured by the above capacitance measurement method. The data of each rabbit before and after blood injection were the data of control group. The experimental steps are as follows:

1. Rabbits were anesthetized with pentobarbital (2%, 1 ml/kg) via ear vein.
2. After anesthesia, 3 ml blood was drawn from the vein of the hind leg.
3. A hole (*d* = 1 mm) was drilled one mm in front of the coronal suture and six mm from the midline. In the head of the rabbit, a tube with a diameter of one mm was inserted into the basal ganglia (the depth of insertion was 13 mm).
4. The blood injection tube and the syringe containing three ml blood was installed on the micro injection pump, ready for blood injection at any time.
5. Placed the rabbit head between the two parallel plates, made the blood injection point at the center of the plate as far as possible, and tried to be close to the plate, but the rabbit head did not contact with the two plates. After half an hour of stabilization, FDC2214 measurement started.
6. Measurement process: static measurement for 9 min before blood injection, then measurement of blood injection process for 9 min, and then static measurement for 9 min after blood injection. Injection blood volume was 2 ml, injection speed was 2 ml/9 min.

The injection points of each rabbit should be consistent as much as possible. Each rabbit was measured for 27 min. The first 9 min data was static measurement without blood injection, the last 9 min data was static measurement after blood injection, and the middle 9 min data was blood injection measurement. In this paper, the capacitance measurement rate is about 15 times per second. Rabbits were euthanized via IV pentobarbital overdose at the end of monitoring.

### Ethics statement of animal experiments

All experimental protocols were approved by the Animal Experiments and Ethics Committee of the Third Military Medical University, the approval number is AMUWEC20201303.

### Signal processing and statistical analyses

In animal experiments, considering that the original signal was affected by respiratory movements plus external interference, a wavelet transform was used to reduce noise. Raw data processing was performed by MATLAB R2015a (Math Works, Inc., USA). Data were presented as mean ±  SD. The Wilcox rank sum test was applied to test the difference in the capacitance value between the injection group data and the control group data in SPSS (SPSS Inc., Chicago, IL, USA). A value of *P* < 0.05 was considered statistically significant.

## Results

### Physical experiment results

The results of Experiment 1 are shown in Figs. 3B and 3C, and the capacitance difference of two solutions at five positions on the *Z*-axis is shown in Fig. 3B. At the position of *h* = 45 mm, the cylindrical vessel was just in the middle position between the two plates. The experimental results show that the closer the vessel is to the two plates, the greater the capacitance difference, the stronger the ability to distinguish the two solutions, and the higher the sensitivity. The sensitivity is the lowest at the midpoint of the distance between the two plates, and the ability to distinguish the two solutions is the weakest. Since the vessel was not filled in by the 9 ml solution, the surface of the solution is about four mm away from the surface of the vessel. When the vessel was placed at two positions (*h* = 10 mm and *h* = 80 mm) close to the upper and lower plate, it is not strictly symmetrical about the middle position between the two plates, so the curve in Fig. 3B are not exactly symmetrically.

The results of the four positions moved along the *X*-axis are shown in Fig. 3C, and *x* = 0 mm corresponds to the center position of the plate. The results show that the capacitance difference of the two solutions decreases and the sensitivity to distinguish the two solutions reduces as the measured position is far away from the center. And the closer to the edge of the plate, the faster the sensitivity decreases. Therefore, the results of Experiment 1 show that for a parallel-plate capacitor, the closer the position is to the plates on both sides, the higher the sensitivity is, and the closer it is to the center of the plate, the higher the sensitivity is.

The results of Experiment 2 are shown in Figs. 3D and 3E. The results are the capacitance changes caused by three different solutions of 9 ml. The results of two positions all shows that the capacitance change of physiological saline is greater than that of glycerin, and glycerin is greater than that of vegetable oil.

The results of Experiment 3 are shown in Fig. 3F, and the measured capacitance is normalized with respect to the initial value. The results show that the capacitance is almost constant before and after injection. With the increase of the injection amount, the capacitance also increases linearly.

### Animal experiments

**Figure 4:**
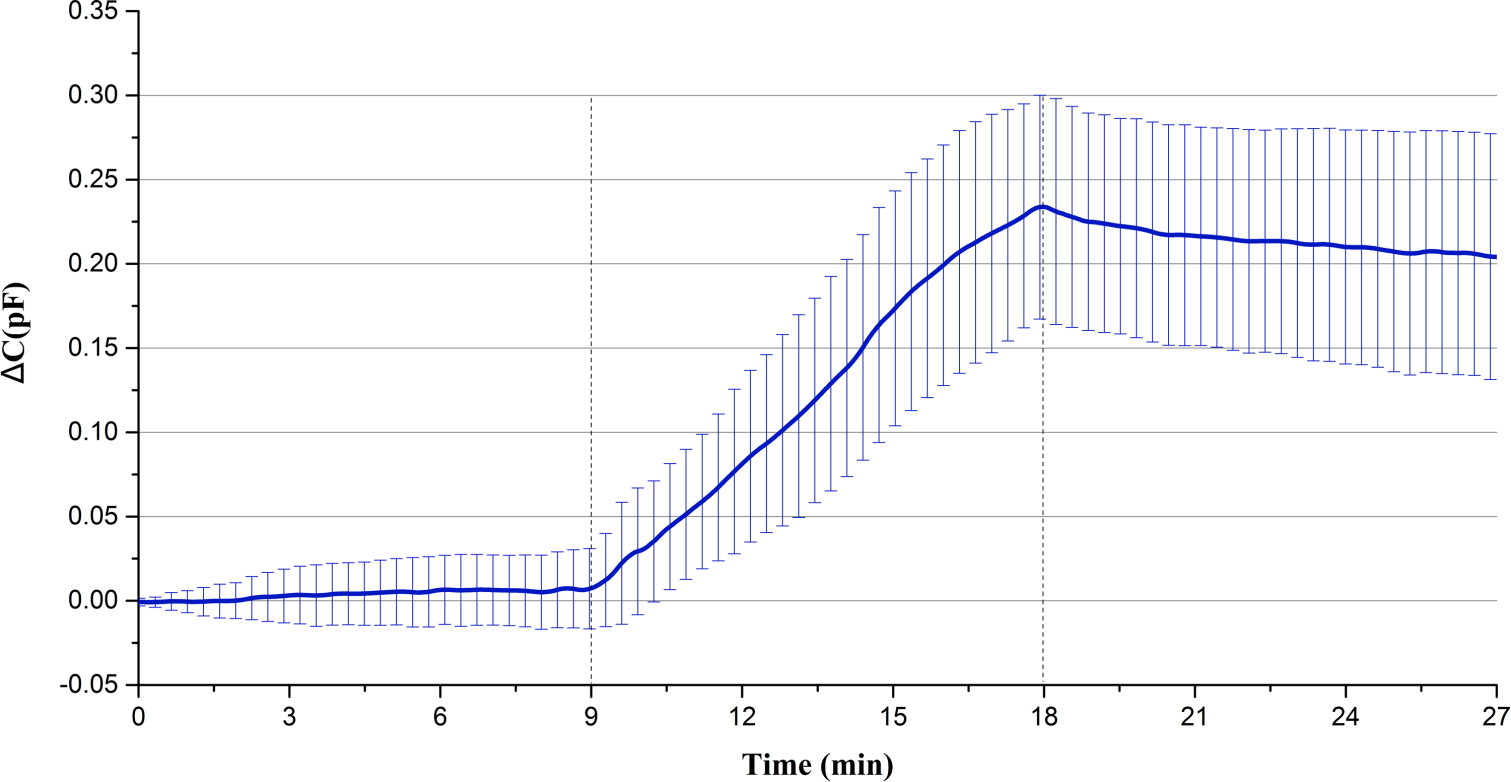
The mean and standard deviation of the capacitance data for fifteen rabbits.

**Figure 5:**
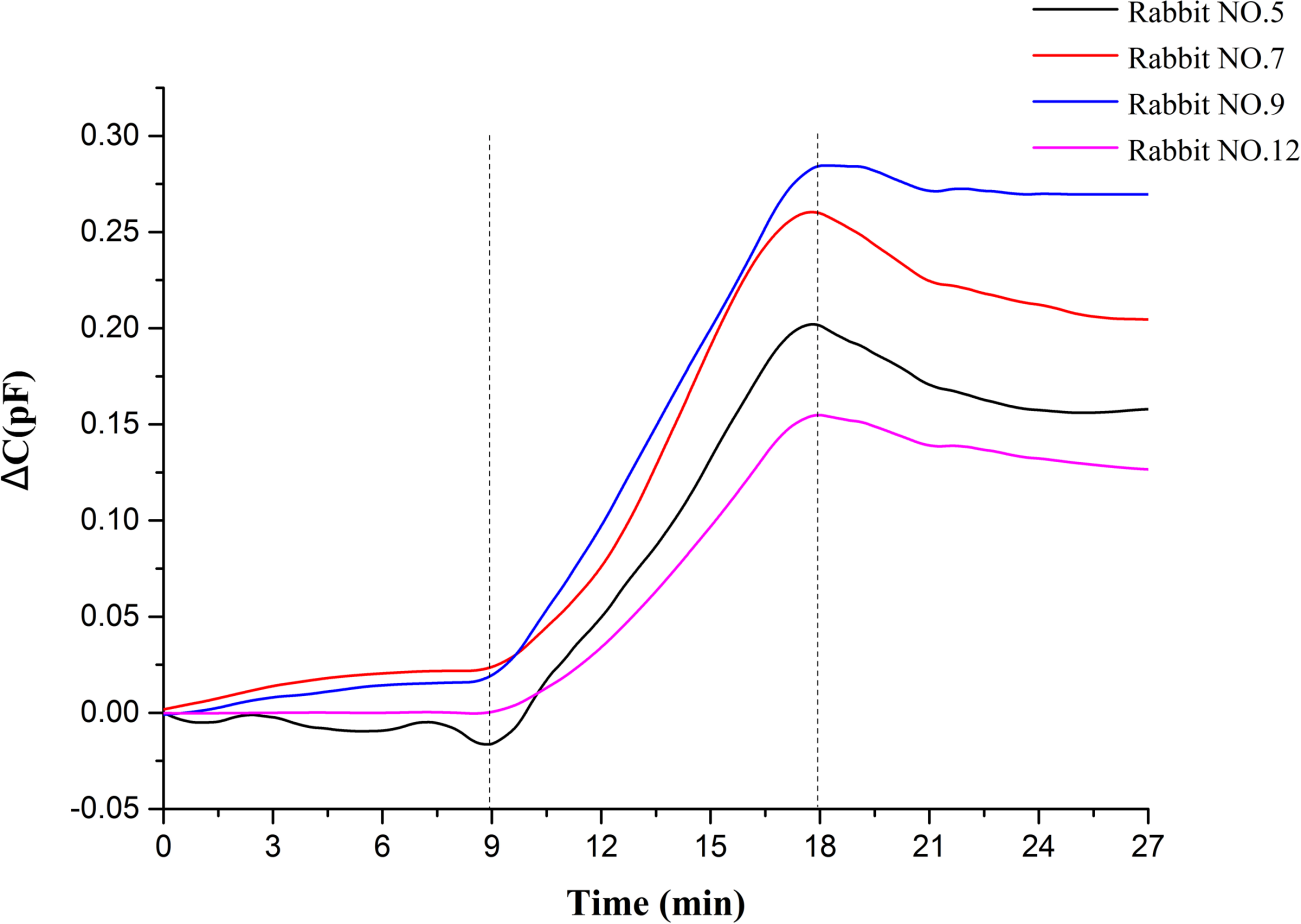
27 min measurement data of four rabbits.

A total of 27 min of data were measured for each rabbit. The data of the first 9 min belongs to the static measurement without injection, the data of the last 9 min belongs to the static measurement after blood injection, and the data of the middle 9 min belongs to the measurement of continuous blood injection. Average the data of each rabbit per second to get the average capacitance value per second in 27 min. Then the data is normalized relative to the initial value, and a wavelet transform is used to remove the heartbeat and respiratory signals and reduce the noise. Finally, the mean value and standard deviation of the data of 15 rabbits at the same time point are calculated, and the results are shown in Fig. 4. Figure 5 displays the raw data of four rabbits. From Figs. 4 and 5, it can be seen that the data of static measurement have little change. In the middle 9 min, the capacitance increases with the increase of blood volume. The statistics indicate that the capacitance increase of blood injection process is much greater than the capacitance changes in anterior non-injection process and the posterior non-injection process (*P* < 0.01), with significant statistical difference. The experimental results are in good agreement with the theoretical predictions. Therefore, it can be proved that the increase of capacitance in the process of injection is really caused by the injected blood.

Secondly, the capacitance increase of each animal is in the 10^−1^pF order of magnitude, so the change of the corresponding resonance frequency of LC circuit is very small. Accordingly, the change of the permittivity of brain tissues caused by such a small frequency change of excitation signal can be ignored. However, the permittivity of blood is always greater than that of other brain tissues at low frequency (<10 MHz) (Gabriel, Lau & Gabriel, 1996), so the increase of capacitance is caused by the increase of blood volume which leads to the increase of the permittivity of the whole brain. So the change of capacitance can reflect the change of hemorrhage volume.

## Discussion

The results of physical experiment 1 shows the sensitivity distribution between the two plates of a capacitor. In a vertical way, the closer to the plates, the higher the sensitivity is, and the sensitivity is the lowest at the midpoint. In a horizontal way, the closer to the center, the higher the sensitivity is, and the sensitivity is the highest at the midpoint. This is corresponding to the electric field distribution between the two parallel plates. The closer to the capacitor plate, the greater the electric field line density, the closer to the edge of the plate, the rarer the field line distribution. Although the results of physical experiment 2 are consistent with the known permittivity of the three solutions. However, the permittivity of glycerol is about half of that of physiological saline, while that of vegetable oil is close to 0. Therefore, the ratio of (ε_saline_-ε_glycerin_) to (ε_glycerin_-ε_*v*_-oil) is about 1:1. But the ratio of (ΔC_saline_-ΔC_glycerin_) to (ΔC_glycerin_*-*Δ*C*_*v*_-oil) is about 1:3. The reason for this may be that the volume of the tested solution is too small (9 ml), and the distance between the two plates is too large (120 mm), so a large space between the two plates is air. In addition, the permittivity of different brands of vegetable oil is also different. It is necessary to use a permittivity meter to measure the accurate permittivity values of three solutions. In physical experiment 3, the permittivity of saline is much larger than that of air, so the capacitance should increase gradually in theory with the increase of saline between plates. The experimental results are consistent with the theoretical predictions. It also shows that the capacitance measurement system can be used to monitor the continuous increase of permittivity. In Fig. 3F, the capacitance has a sudden change at the beginning of injection, which is caused by the vibration and displacement of the solution conduit when the injection is started.

The animal results show the capacitance increases approximate linearly with the increase of blood volume, but is almost constant before and after injection. So the change of capacitance can reflect the change of hemorrhage volume. In Fig. 5, the data curve of each rabbit after blood injection have a small drop, which is not like the curve of a horizontal straight line before injection. This may be because the blood injected was added with heparin. Heparin can prevent the blood from clotting and make the blood be diluted. The injected blood cannot clot in the rabbit brain. It will flow and be porous. The blood volume in the brain increased continuously during the continuous injection, and once the injection stopped, the blood would disperse because of the free flow. Different positions have different sensitivity, which results in a small drop in capacitance.

Although the capacitance value of each rabbit increases with the increase of blood injection, but there are some differences between each rabbit. The reasons may be as follows: (1) individual differences between animals. (2) The relative position between the parallel plate and the rabbit head was not absolutely identical for all animals. (3) It was difficult to keep the location of injection point and depth consistent for all animals. (4) The depth of anesthesia was different in each animal. (some animals had convulsions and restlessness due to shallow anesthesia). (5) The blood injected with heparin would flow randomly in the rabbit brain, and there are different sensitivities in different positions between the plates. These two reasons also lead to the difference of capacitance increment.

## Conclusions

According to the fact that the permittivity of blood is much larger than that of other brain tissues, and the relationship between the change of permittivity and the capacitance is in direct proportion, we designed a parallel-plate capacitor to clamp the rabbit head, and by measuring the capacitance of the parallel-plate capacitor to evaluate the change of intracranial hemorrhage volume in rabbit brain. Physical experiments show that the parallel-plate capacitor can distinguish three kinds of liquids with different permittivity and can monitor the continuous increase of permittivity. In addition, the experiment shows that in the plane parallel to the polar plate, the closer to the center of the plate, the higher the sensitivity is. In the direction perpendicular to the plate, the closer to the polar plate, the higher the sensitivity is. The results of physical experiments provide guidance and strong support for animal experiments. The results of animal experiments show that there is no significant change in capacitance value without blood injection. However, the capacitance increases with the increase of blood volume. The change of capacitance caused by blood injection is much larger than that of the static measurements (*P* < 0.01). The results of animal experiments are consistent with the theoretical predictions. The experiments prove that it is feasible to evaluate the amount of bleeding by measuring the capacitance of the head. However, the results are not well in correspondence with each other, which may be caused by individual differences of animals, operation and measurement errors. In addition, the blood injection model in this paper is different from the actual bleeding model. In this paper, the blood is added with heparin, will not coagulate, is not easy to form hematoma, and will flow in the brain at will. But the actual cerebral hemorrhage is easy to form hematoma. Therefore, it should get a better result to measure the actual hematoma. The next step is to improve the animal experimental model, further explore and improve the measurement methods. In general, the experimental results of this paper prove the feasibility of measuring intracranial hemorrhage by a parallel-plate capacitor.

##  Supplemental Information

10.7717/peerj.10583/supp-1 Supplemental Information 1 Raw data from Physics Experiment 1

10.7717/peerj.10583/supp-2 Supplemental Information 2 Raw data from Physics Experiment 2

10.7717/peerj.10583/supp-3 Supplemental Information 3 Raw data from Physics Experiment 3

10.7717/peerj.10583/supp-4 Supplemental Information 4 Raw data from Animal Experiments

## Additional Information and Declarations

### Data Availability

The following information was supplied regarding data availability: Data collected from 15 rabbits and the raw data of physical experiments are available in the Supplemental Files.
